# Explaining the Relationship Between Minority Group Status and Health Disparities: A Review of Selected Concepts

**DOI:** 10.1089/heq.2018.0035

**Published:** 2019-03-07

**Authors:** Judy H. Ng, Lauren M. Ward, Madeleine Shea, Liz Hart, Paul Guerino, Sarah Hudson Scholle

**Affiliations:** ^1^ National Committee for Quality Assurance, Washington, District of Columbia.; ^2^ Columbia University Mailman School of Public Health, New York, New York.; ^3^ Health Management Associates, Washington, District of Columbia.; ^4^ American Hospital Association, Chicago, Illinois.

**Keywords:** epigenetics, health disparities, life course, minority stress, resilience

## Abstract

**Purpose:** There is growing concern that value-based payment for health care may disadvantage health care organizations that serve populations with social risk. In the broader investigation of social risk factors, including income, education, neighborhood deprivation, and other risks, the focus on race and ethnicity as a risk factor for disparities in health and health care has diminished. Understanding the independent contribution of minority group status is critical to this discussion. This narrative review discusses four concepts—minority stress, resilience, epigenetics, and life course—that may help explain the contribution of minority group status and its association with health disparities.

**Methods:** We briefly describe each concept and the supporting evidence.

**Results:** Our results indicate that all four concepts have potential relevance for understanding and addressing health disparities. The life course perspective emphasizes the importance of understanding explanatory mechanisms and factors that contribute to health—including biological, physical, and social factors—over a person's life span. Both minority stress and resilience may influence health in either a negative or positive manner that potentially underlies health changes. Exposure to these factors and others may interact with and modify epigenetic regulation—biological processes that impact how our genes are expressed. This may increase the risk of disease and negative health outcomes, particularly among groups that may be at disproportionate risk because of social circumstances and environmental exposure over the life course.

**Conclusion:** Despite these concepts' relevance, more research is needed to assess how they may explain the relationship between minority status and disparities in health. Such evidence is needed to focus interventions and to inform the design of delivery and payment models that can spur actions to reduce disparities.

## Introduction

Although a large body of literature has established persistent disparities in health and health care for racial and ethnic minorities, another important discussion has begun in tandem: whether observed disparities associated with race and ethnicity may represent socioeconomic position or other factors that are “less easily measured.”^1^ Most recently, federal policies such as the Improving Medicare Post-Acute Care Transformation Act of 2014 have generated national attention and investigation into the role that social risk factors—including race and ethnicity,^2^ low income, education, and other factors of socioeconomic disadvantage—may play in health outcomes, and how to account for this in quality measurement and value-based payment so that organizations serving at-risk populations are not disadvantaged.^3^

Consequently, federal agencies and national organizations are examining the role of social risk factors on quality measurement and payment, particularly in Medicare, including the Office of the Assistant Secretary for Planning and Evaluation,^4^ the Centers for Medicare & Medicaid Services,^5^ and the National Academies of Sciences, Engineering and Medicine.^6^

Much of this recent work focuses on social risk factors related to income, education, and other risks, with less attention paid to race and ethnicity. However, minority status based on race and ethnicity or other social risks described above is often observed together. For example, researchers have highlighted the impact of environmental and social factors that correlate with biological changes in racial and ethnic minority groups,^7^ and the disproportionate representation of racial and ethnic minorities among the poor.^8,9^ There is also evidence of a relationship between exposure to environmental toxins and experiences of stress, with increased risk of health conditions such as cardiovascular disease and cancer among racial and ethnic minority adults.^10–12^ Similarly, the literature documents the relationship between racial discrimination and health.^13–17^

Differing concepts have emerged to explain these observed relationships—including minority stress, resilience, epigenetics, and life course—and the potential link between racial and ethnic status and health disparities.^18–21^ These concepts are of special interest because they are alluded to in the public health literature—yet, are often referenced separately, with limited discussion of their potential relationship to one another and to health disparities.

Understanding these concepts and how race and ethnicity—and other social risk factors—contribute to health and quality-of-care outcomes is critical to current discussions. It can improve our ability to parse the role of different risk factors in health and health care outcomes, to understand underlying mechanisms, target interventions and modifiable pathways, and inform payment or delivery models that spur reductions in disparities.

Although prior systematic reviews address the role that minority stress, resilience, epigenetics, and life course may play in the health of racial and ethnic or other minorities, few explicitly assess whether these concepts help explain disparities in health between populations with differing levels of advantage. Furthermore, despite emerging literature that considers these concepts jointly,^22–25^ there is limited research and conceptualization explaining how they interact and contribute to disparities.

The purpose of this narrative review is to assess existing systematic reviews and consider the strength of evidence regarding how concepts of minority stress, resilience, epigenetics, and life course may independently or jointly explain how minority status affects health and health care disparities in ways not accounted for through other socioeconomic factors. We assess how each concept is operationalized, key health issues and disparities associated with each concept, the strength of evidence, and competing or complementary concepts. We also explore how the four concepts may work together. The synthesis of this information may help focus on interventions and inform the design of delivery and payment models to reduce disparities.

## Methods

We conducted our literature review between 2016 and 2018. We first conducted a preliminary search in PubMed to identify literature that provided an overview of minority stress, resilience, epigenetics, and life course concepts, and their influence on minority group status-related disparities. Search terms addressed minority group status, health outcomes and disparities, and multiple socioeconomic position-related variables (details in Appendix Tables A1 and A2), resulting in 3239 articles. We also consulted two subject matter experts on epigenetics, minority stress, and life course for input on the topics and further articles, resulting in 3250 articles.

Next, we searched within initial results to obtain information on the strength of evidence related to each concept and its impact on minority group health disparities. We excluded 3004 articles that were duplicates; non-English, nonhuman subject articles; published before 2001; and not systematic reviews or meta-analyses addressing each concept's strength of evidence. Among the remaining 246 articles, published between 2001 and 2018, we focused primarily on U.S.-based systematic reviews and meta-analyses addressing health disparities among racial and ethnic minority groups.

**FIG. 1.**
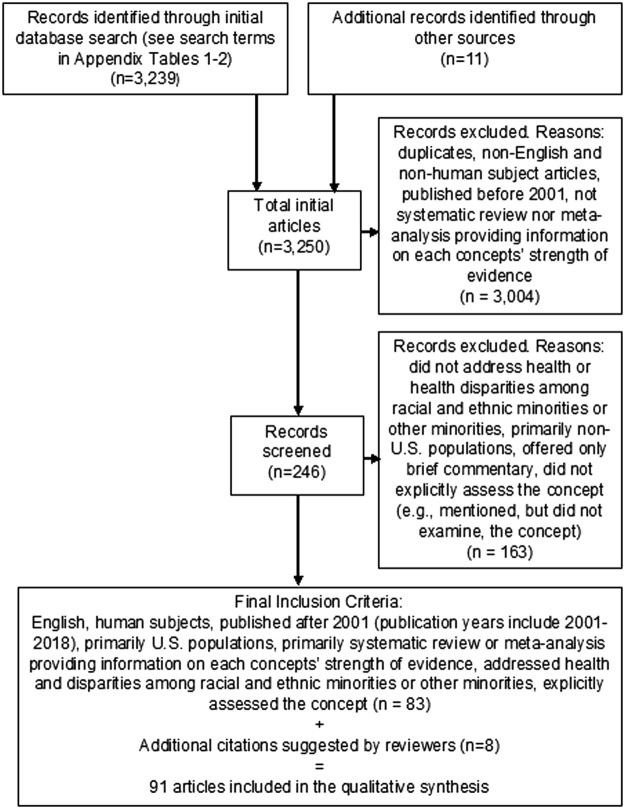
PRISMA flow diagram.

We excluded articles that did not address health-related outcomes or outcomes for minority populations, did not assess the concept of interest (e.g., mentioned, but did not explicitly examine, resilience), or offered only brief commentary. We turned to individual studies if they provided recent evidence not included in systematic reviews. We identified 83 references based on these criteria, with an additional 9 suggested by reviewers, totaling 91 references for this review. Figure 1 depicts this process.

## Results

**FIG. 2.**
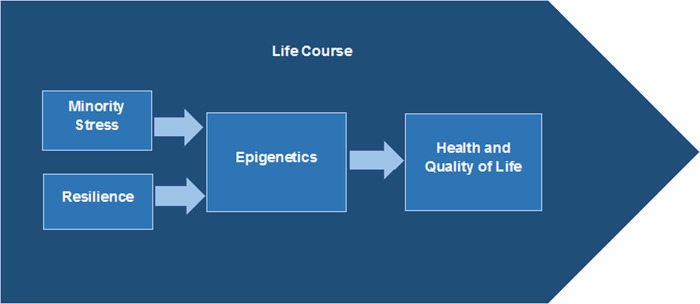
How concepts might work together.

Although more systematic reviews are needed, evidence suggests that the concepts of minority stress, resilience, epigenetics, and life course are relevant for understanding and addressing the association between racial and ethnic minority status and health disparities. We describe findings for each concept in the following sections (summarized in Table 1), followed by a description of how these concepts may fit together (Fig. 2):

**Table 1.** Summary of Minority Stress, Resilience, Epigenetics, and Life Course Concepts

Concept	Minority stress	Resilience	Epigenetics	Life course
Definition	The experiences of psychological stress or heightened physiological responses that result from chronic or acute experiences of unfair treatment and abusive behavior related to one's belonging to a stigmatized minority group (e.g., prejudice and discrimination).	Although lacking a universal definition, resilience is the concept of adapting well in the face of adversity, trauma, or threat. Common elements include recovering or “bouncing back” from adversity, rising above adversity, an adaptation, or adjustment process, or absence or lower incidence of mental health issues after adversity.	Changes in gene expression regulated by the epigenome—biological process that direct and modify DNA expression. These epigenomic modifiers (also known as regulators) may be altered by social, cultural, psychological, and physical environment exposure.	A framework that considers how human development is shaped over one's life, with periods of importance (childhood, adolescence, midlife, and older age), and emphasizes the importance of experiences at each life stage, and of the individual's role as an interactive part of a larger social, cultural, and physical environment, on their health.
Mechanism of action/factors of importance	Two potential pathways for impact on health: (1) by activation of a prolonged stress response that affects health (e.g., hypertension) or (2) by affecting health behavior as a coping response (e.g., smoking).	Resilience-related protective factors include resources such as social support and family coherence, which facilitate resilience. *There may also be vulnerability factors such as exposure to trauma that moderates the effect of resilience on outcomes*.	Process, including DNA methylation, histone modification, and RNA silencing. Examples of factors that may influence epigenetic regulation include maternal behaviors during pregnancy, paternal obesity, social interaction and behavior, diet and exercise, drug abuse, and environmental chemicals. *Some of the evidence to support this is based on animal studies*.	Social, cultural, physical, and other factors that interact or accumulate over time to affect health. *Examples of research using the life course framework include studies examining how adverse childhood events affect health later in life.*
Measurement of concept in studies	Self-reported episodes of discrimination, or perceived discrimination. *Standardized assessment tools include The Perceived Racism Scale*, *The Everyday Discrimination Scale*, *The Schedule of Racist Events*, *and The Experiences of Discrimination measure*.	Multiple approaches to assessment, including measures of “stable” personality traits, dynamic processes, resources, or protective factors that facilitate resilience, or types of “attitudes.” *Standardized assessment tools include Connor-Davidson Resilience Scale;and Resilience Scale.*	Level of epigenetic regulation in specific gene regions, or of specific genes associated with diseases.	Life events that may be relevant when considering health disparities depending on the period of focus. For example, measures of adverse childhood events, environmental exposures *in utero*, family dysfunction, or extreme childhood deprivation to examine relationships between social factors early in life and long-term effects on health.
Strength of evidence for concept to explain disparities	Individual studies suggest an association between minority stress and health disparities. Systematic reviews did not demonstrate this link. Systematic reviews focused on the association between discrimination and stress, and discrimination and health outcomes within each minority group.	Multiple individual studies have examined evidence of the role of resilience in moderating or explaining health disparities in vulnerable groups. However, systematic reviews and meta-analysis of resilience have not examined evidence between minority groups compared to nonminority referent groups.	Existing literature has sought to establish evidence of regulation of specific genes for health outcomes such as cardiovascular disease, metabolic disease, pre-term birth, and cancer. However, few systematic reviews and meta-analyses have examined the potential link to disparities in health or health care based on racial or ethnic group status or other social risk factors.	There is compelling evidence to support the life course perspective, mostly focused on early life socioeconomic conditions and adult health outcomes. However, evidence regarding causal mechanism is limited.
Examples of adult health outcomes investigated	Mental health, cortisol levels, blood pressure, and general physical well-being.	Substance abuse, mental health; diabetes and cardiovascular disease; and cellular aging.	Cardiovascular disease, metabolic diseases, cancer, pre-term birth, and chronic disease.	Cardiovascular disease and other chronic disease (e.g., diabetes and cancer), and quality of life (e.g., SF-36 mental component scores).
Related concepts	Related concepts consider “weathering,” allostatic load, and acculturative stress.	Lack of a universal definition has resulted in multiple approaches to defining and operationalizing resilience.	Competing concept is the hypothesis that actual genetic variation between populations explain health differences.	Competing concept is that individuals have responsibility for their own health and health-related behavior, rather than accounting for the impact other factors may have on individuals' health and health behavior.
Groups commonly addressed in the literature	African American; European American; Hispanic/Latin/o American. Increasingly, studies address Asian Americans, other indigenous populations, immigrants, and other minority groups like sexual and gender minorities.	Multiple vulnerable populations of interest (e.g., racial and ethnic groups, low-income populations, immigrant populations, and sexual and gender minority); teenagers are a common focus in resilience studies.	Racial and ethnic subgroups: African Americans, Hispanics, and Whites.	Often examines how adverse childhood events affect health outcomes later in life—some articles look at stratification by race and ethnicity group.
Adult health outcomes commonly addressed in the literature	Poor mental health, positive mental health (e.g., self-esteem), general physical well-being, hypertension, obesity, and adverse birth outcomes.	Substance abuse, mental health, diabetes and cardiovascular disease, and cellular aging.	Cardiovascular disease, metabolic diseases, pre-term birth, and general reference to chronic disease.	Cardiovascular disease and other chronic disease (e.g., diabetes and cancer), quality of life (e.g., Short Form-36 mental component scores).

### Minority stress

Minority stress describes the chronic stress resulting from experiences or perceptions of unfair treatment or abusive behavior based on belonging to a stigmatized minority group. The minority stress model is a framework for conceptualizing how experiences unique to minority groups—prejudice and discrimination, in particular—confer chronic psychological stress and heightened physiological responses that impact mental and physical health over time.^11,26–28^

There are two conceptual pathways through which discrimination may affect health: through activation of an emotional and physiological stress response and by impacting health behavior. In the first pathway, discrimination acts as a stressor that adversely effects emotional (e.g., anger) and physiological response (e.g., increased blood pressure) and, when activated frequently over time, strains biological systems and increases risk of poor physical and mental health outcomes.^20,27,29–31^

In the second pathway, discrimination has an impact on health behavior, “either directly as stress coping, or indirectly, through self-regulation.”^20^ For example, discrimination may result in unhealthy behavior (e.g., smoking or drinking as coping mechanisms) or failure to participate in healthy behavior (e.g., disease screening or management), which has clear links to health outcomes.^27^ Some research has shown that experiencing discrimination can decrease self-control resources and increase the risk of poor health behaviors, but other evidence did not support a clear link between discrimination and poor health behavior.^20,27,32,33^

Although few systematic reviews or meta-analyses explicitly examine the relationship between minority stress and health disparities (a gap confirmed by a subject matter expert), we found research assessing the association between discrimination and stress, and between discrimination and mental and physical health outcomes. For instance, evidence demonstrated that experiences of discrimination caused a variety of psychological and physiological stress responses.^27,28,34^ Some systematic reviews suggested a link between discrimination and health outcomes, but the relationship was not always significant or consistent.^11,27,28,35^ More research identifying evidence to support the link and conceptual pathway between minority stress and health disparities is needed.

The literature also touched on complementary concepts, in addition to minority stress, for example, the concept of weathering, which refers to deteriorating health from “the cumulative impact of repeated experience with social or economic adversity and … marginalization” (i.e., repetitive stress may influence health outcomes or disease prevalence).^36^ A broader, but related, concept is allostatic load, an assessment of the body's physiological stress response, which may be useful to quantify the biological basis for the link between discrimination and health.^37^

The literature also addressed related forms of stress, such as acculturative stress, which refer to the tension and anxiety that accompany efforts to adapt to the “orientation and values of dominant culture [i.e. social group]” and which have been linked to disparities in hypertension and mental health among minority groups.^38,39^

### Resilience

Resilience generally refers to positive adaption in the face of negative life experiences or adversity—including threats and trauma (e.g., adverse childhood events).^19,40–42^ While a universal, precise definition is lacking, common characteristics include recovering from or rising above adversity, an adjustment process, or an absence or lower incidence of mental health issues resulting from adversity.^43^ The emphasis is on how individuals use resources to negotiate, “bounce back,” or adapt to adversity.^44^ The potential pathway between resilience and health outcomes occurs through moderation of the relationship between the “antecedent” event—exposure to adversity—and eventual health.^19^

Factors that may moderate this pathway include protective factors (family connectedness, social support, religious involvement, and diversity of friendships) that facilitate resilience, and vulnerability factors (poverty, interpersonal violence, household dysfunction, heightened vigilance to threat, and parental mental illness) that may amplify the negative impact of adversity on health.^19,45–49^

Resilience is of special interest in health disparities research because it seeks to identify how people achieve positive outcomes in the face of adversity (e.g., avoiding negative outcomes associated with adversity such as poor health, and coping with trauma), rather than focusing only on an explanatory mechanism for poor health outcomes.^44,50,51^ Understanding resilience can facilitate development and implementation of more sensitive, effective strategies to foster positive adaptation to decrease health disparities.^19^ Promoting resilience as early as possible may be as important as other types of interventions to address problems among at-risk individuals.^19^

Lack of a universal definition of resilience has led to multiple approaches to operationalizing this concept.^43^ Inconsistencies make it challenging to compare studies in systematic reviews and meta-analyses or to comment on the overall strength of evidence regarding the impact of resilience on health and health disparities.^43,52^

Most systematic reviews of resilience examined its impact on various health indicators for people with minority status, but did not explicitly address or assess *disparateness* in health between minority versus referent (usually more advantaged) populations—that is, there was no comparison between populations of differing advantage. However, we identified individual studies of racial and ethnic minority groups, suggesting that resilience might affect a variety of health outcomes, including mental health, diabetes, cardiovascular disease, and cellular aging.^23,45,53–55^

The lack of a universal definition has also fostered complementary and competing concepts to explain resilience,^43^ from describing it as a set of individual traits to protective factors or adaptive processes.^43,52^ An important competing concept in the literature is that resilience is only “skin deep.”^50^ For example, there is evidence that despite outward-seeming “successes” (e.g., academic or social competence) in resilient individuals experiencing stressors or adversity, attaining such success takes an internal physiologic toll, including higher rates of immune cell aging and cortisol levels.^23,50^ These studies suggest a cost to maintaining self-control and competence in the face of adversity.

### Epigenetics

Epigenetics is the study of changes in gene expression regulated by the epigenome—the set of biological processes that control and modify the expression of DNA.^56–58^ Some evidence indicates that epigenomic regulators can be altered by exposure to social, psychological, physical, and environmental factors, starting *in utero* and continuing from infancy to adulthood (although some evidence is based on animal models).^7,57,59^

The resulting gene expression is a reflection of gene–environment interaction over time.^7^ The impact of these factors may not be expressed immediately, but may have downstream effects that pre-dispose individuals to disease and affect health later in life, potentially into future generations.^7,18,60–62^ This hypothesis has been used as a basis for the Development Origins of Health and Disease (DoHAD) model, which describes how early life experiences shape adult health through epigenetic modifications that can alter the long-term risk of disease.^18^

Epigenetic findings are relevant to explaining the incidence of disease, because abnormal gene expression underlies many human diseases.^12^ Health disparities between populations are thought to be the result of the complex interplay between biological, social, environmental, and behavioral factors.^18^ Because minority groups may be at disproportionate risk as a result of social circumstances and environmental factors that influence epigenetic modifications over time, some researchers are exploring the role that abnormal gene expression plays in observed racial and ethnic disparities in health.^12,18,63,64^

Few literature reviews and meta-analyses examined differences in epigenetic regulation between minority groups and referent groups; many focused on one minority group.^65–68^ We did, however, find individual studies that investigated differences between groups or sought to establish evidence of epigenetic regulation in groups with social risk factors, even if they did not explicitly address health disparities (i.e., comparing nonminority, more-advantaged groups with minority, less-advantaged counterparts in the same assessment). Studies generally focused on racial and ethnic minorities for a variety of health outcomes (e.g., cardiovascular disease, metabolic diseases, cancer, and pre-term birth).^69–72^

We also found that researchers considered observational evidence and looked to adult levels of epigenetic regulation to demonstrate the epigenetic basis for disease and disparities.^12^ Reviews indicate that even after accounting for access to care and genetics, epigenetic regulation “levels” differ between racial and ethnic groups, suggesting that epigenetic regulation may be a contributing factor to disparities.^12^

Several competing concepts are used to explain observed racial and ethnic disparities; one is that genetic variation between racial and ethnic groups—not epigenetic differences—accounts for differences in health outcomes. Studies have suggested that genetic factors may put specific minority groups at higher risk for certain health outcomes and explain disparities between and across minority groups.^7,12^

However, critics argue that considering genetic differences alone does not account for observed differences in health outcomes, even after accounting for social risk factors, and ignores the evidence that social factors affect health over a lifetime.^63^ One area where this is most evident is in studies of monozygotic twins; epigenetic differences are observed over the life course, indicating the continuing role of epigenetic regulation in gene expression.^18^

### Life course

The life course concept proposes that human development is shaped by experiences over phases in our lives, with particular life-cycle periods of importance (e.g., fetal, childhood, adolescence, midlife, and old age).^73^ This perspective may be used to understand how experiences in various parts of life can bridge to and affect later health.^21,74^ Life course emphasizes the importance of experiences, or their accumulation, at each life stage, not only at a point in time. It also considers the role of the larger social and physical environment in producing health, not the individual as an isolated unit.^73–77^

Life course concept derives from psychology, biology, sociology, and public health.^76^ Researchers have identified a subset of concepts within life course that may be especially relevant in addressing health disparities based on race, ethnicity, and socioeconomic status: (1) sensitive periods (events and experiences have a more pronounced effect on health if they occur in sensitive periods [e.g., childhood]); (2) the accumulation effect (does not favor a period; proposes that events and experiences have a cumulative effect on health over time); and (3) linked lives (individuals are interdependent; events affecting someone in a network may affect others in the network).^21,43,76–82^ (Refer to Table 2 for specific examples.)

**Table 2.** Specific Life Course Concepts with Relevance for Health Disparities

Life course concept (also referred to as Life Course Models)	Example(s)
*Sensitive period(s):* Under this concept, certain events or experiences are considered to have a more pronounced effect on health if they occur within select periods in life.^77^	*Examples*: (1) Malnutrition in early childhood has a greater effect on health than malnutrition at other periods in life. (2) Exposure to higher income neighborhoods in earlier childhood (pre-age 13) has a greater effect on positive outcomes in adulthood (e.g., higher earnings) than the same exposure in later childhood (after age 13).^80^
*Accumulation*, *or cumulative impact:* This concept does not favor a particular period for which events or experiences have a more pronounced impact—rather it proposes that the effects of events/experiences “add up” over time and have cumulative effect on health (including a dose–response effect), beyond the unique explanatory effect of any one event/experience or factor.^21,76,79^	Exposure to adverse, socioeconomic experiences in childhood and adulthood (e.g., poverty, urban violence, ongoing/perceived discrimination based on race/ethnicity, disability, sexual and gender minority status. or other social characteristics) over time have a cumulative, dose-effect, impact on health outcomes.^21,76,79^
*Social pathways (or* “*age-patterned exposures*”*):* Refers to pathways (or the types of exposures) that individuals follow through the life course, which can be influenced by social settings and historical events. Throughout the life course, individuals may move through different social settings and experience variations from one setting to another. It is possible for the influence of an event in childhood to be attenuated by other events later in life.^77,79^	Racial and ethnic minorities may be exposed to discrimination in certain settings (e.g., school, work), and the prevalence of discrimination may vary over the life course. Discrimination in one setting (school) may differ from another (work), but exposure to one may reverberate and further increase the effect of exposure later.^77,79^
*Linked lives:* Individuals are interdependent, events affecting one person may affect another person in the same network.^76,77^	*Examples:* (1) Discrimination based on race and ethnicity (or other social characteristics, such as disability or sexual and gender minority status) may have deleterious effect if it is experienced by someone in an individual's network, even if the individual does not himself or herself experience discrimination.^76,77^ (2) Behavioral traits related to obesity, a condition whose prevalence has been especially noted in minority groups, may be spread through “social ties”—longitudinal evidence has established that if a person has a friend who is obese, that person's chances of becoming obese also increases.^81^
*Cohort effect:* Individuals belonging to different birth cohorts may be affected differently by historical and social events.^77^	Racial disparities in infant mortality diminished in the period after the Civil Rights Act.^77^
*Intergenerational/transgenerational effect (also discussed under the concept of “historical trauma”):* Events—particularly traumatic, discriminatory and/or “racialized events”—experienced by one generation (e.g., Wounded Knee Massacre against the Lakota people, slavery, the Holocaust) may be felt by following generations.^78,79^	*Examples:* (1) Research on government policies toward the Lakota people demonstrate disrupted culture-based protective factors, including grieving processes. Among multiple Native American populations, frequency of thinking of losses associated with historical traumas is as follows: associated with distressed feelings, mediates effects of perceived discrimination and alcohol abuse, and contributes distress independent of other stressors.^78^ (2) Evidence from the Dutch famine cohort indicates that mothers who experienced malnourishment had children with epigenetic changes in genes involved in growth, diabetes, and obesity.^7,18^

In general, life course research examines a range of social, physical, and other factors that may confer advantage or disadvantage in their effect on health. Studies have investigated the impact of a variety of factors (including racism and discrimination) on health inequity over the life course, but the complexity of the impact is considered underresearched.^77^

There is compelling evidence to support the life course perspective. Epidemiological and other evidence have linked experiences in early life—particularly low birth weight, socioeconomic conditions, or adverse childhood events—to a range of health outcomes in later life.^21,83^ Longitudinal evidence from other disciplines also demonstrates the importance of early childhood experiences on later health.^80^ However, there is less evidence of the mechanisms explaining how these factors act to produce or influence health—a situation that has been exacerbated by ongoing challenges in measuring relevant exposures and factors over the course of a lifetime.^84^

The main competing concept to this perspective is the implicit view that health is the result of individual actions and behaviors and is not impacted by events—often beyond an individual's control—over a lifetime.^21^ It suggests that individual behaviors alone, not external influences such as discrimination or stress, are responsible for health. However, this concept has been criticized and the literature has observed that public health efforts targeting only individual behaviors, without accounting for social and environment factors, may have exacerbated disparities. This includes failure to acknowledge that socially disadvantaged groups have greater obstacles to adopting healthier behaviors and thus experience fewer health improvements than advantaged groups.^21^

Another critique is that life course studies assessing disparities focus on adult health and pay little attention to child health.^85^

### How do these concepts work together?

The four concepts—minority stress, resilience, epigenetics, and life course—have potential relevance for addressing health disparities. All influence health in different ways (Fig. 2).

Life course may be considered an organizing framework for synthesizing information on factors that influence health.^76^ It is helpful for understanding and addressing health disparities because it “directs attention to the role of … social and physical context along with biological factors over time” in shaping health, and because “social and physical contextual factors underlie socioeconomic and racial and ethnic disparities in health.”^21^ This perspective may be useful for connecting other concepts—epigenetics, minority stress, and resilience—and could suggest how they interact and influence health.

The literature posits a variety of factors and mechanisms that might interact to influence health.^78^ Among these, minority stress and resilience can be considered two sides of the same coin. Minority stress can *adversely* influence health over time; resilience may interact with environmental factors to *positively* affect health.^43^

There is growing understanding of epigenetics as a potential explanatory mechanism at the biological level that underlies the processes shaping health.^82^ Environmental, social, and other factors—including minority stress and resilience—may influence health through epigenetics and other biological processes.^86^ This concept has benefitted from new reviews and studies examining biological mechanisms and their role in the association between minority status (including low socioeconomic status) and health disparities.^87,88^

Under the life course concept, exposure to different factors and their interactions over a lifetime may affect biological processes and contribute to disease prevalence and health disparities.^77^ The DoHAD model (detailed in the epigenetics overview) is one example.^18^ Despite the paucity of empirical studies that directly assess these exposures and relationships over a lifetime, recent efforts provide promising empirical support, including studies using data from a larger number of points in the adult life course.^88^

As evidence emerges, these concepts and their interrelation may shift. Recent literature includes assessments of multiple concepts within the same study, for example, examining life course and resilience simultaneously, and the intersection between epigenetic aging, minority stress, and resilience.^22–24,89^

A recent review explicitly assessed the intersection of multiple minority groups (e.g., being a racial and ethnic minority, and a sexual and gender minority) and the potential role of these concepts.^90^ It may be relevant to consider complementary concepts to understand how they might interact and contribute to health disparities. The concept of weathering may be useful in understanding how minority status impacts gene expression and health over a lifetime, and the DoHAD hypothesis is a relevant conceptual model for considering the impact of epigenetic regulation over a lifetime.^18,36,91^

## Discussion

Our review examined current evidence on the relevance of minority stress, resilience, epigenetics, and life course for addressing health disparities—focusing on minority status as a proxy for other factors. Several themes and implications emerged.

First, while all four concepts have relevance for understanding and addressing health disparities, gaps reduce their utility for health policies and programs—including the lack of universal definitions and agreement on measurement approach; evidence to support specific causal mechanisms; comparative literature (due to variation in measures across studies); and systematic reviews that examine these concepts through the lens of health disparities.

Second, when considering health disparities, it is important to examine a range of influencing factors. A single factor—even a cluster of factors—may contribute to disparities, but is not enough to explain them.

Third, it is important to look beyond an individual's current circumstances and consider experiences in earlier periods of life and throughout life—even intergenerational experiences—to truly understand health disparities and improve health outcomes.

Fourth, experiences of discrimination may impact the health of minority groups. Belonging to a minority group may be a proxy for the presence of minority stress that negatively affects health, especially in the long term. Any approach to addressing health disparities must account for minority status or miss a potential factor that can amplify disparities.

Fifth, this research may be useful in developing disparity-focused interventions. For example, resilience recognizes that some people do well despite adversity. There is an opportunity to foster resilience among minority groups as our understanding of resilience and resilience-related interventions' increases. Research suggests that early intervention is important and relevant—to resilience, and to life course and epigenetics. Identifying coping strategies that bolster resilience in minority groups can inform interventions.

Sixth, this research suggests opportunities to improve health outcomes by incorporating elements that increase resilience, target life course harms, and reduce minority stress—which can all impact epigenetics. As value-based payment models for care are implemented, there should be efforts to assess quality, experiences, and outcomes by race, ethnicity, and other factors. This can promote understanding of how policy changes may have differential impacts among minority groups and suggest ways to adjust programs to reduce disparities.

Given the life course effects of disproportionate harms among racial, ethnic, and other minority groups, innovations that improve health during key life course stages may have a magnified impact; for example, addressing nutrition during pregnancy, infancy, and childhood. Health care payers could test approaches to alleviating minority stress—or increasing resilience—as mechanisms to reduce health disparities. There is also a need to better understand how value-based approaches to health care can incorporate such efforts—including how payers could reimburse and incentivize disparities reduction efforts (e.g., reimbursing Medicare and Medicaid providers for social services could address disparities on a federal and statewide scale).

Despite the potential role of these concepts, their explanatory power is not without controversy. Epigenetic science provided early evidence of the underlying biological mechanisms that regulate genetic expression leading to health disparities, but its contribution to explaining them is open to debate^18,76^—particularly with regard to accounting for disparities by racial minority status, which is a social construct rather than a biological fact. As evidence continues to build on how social, physical, and biologic factors interact over time, and on the mechanisms and factors underlying these interactions, there may be shifts in concepts and how they relate to each other, and their role in the persistence of health disparities for minority groups.

Because there are gaps in the literature, this review is subject to the limitations stated above; thus, it is difficult to confirm each concept's role in explaining the association between minority group status and health disparities. Furthermore, this review did not explicitly address discriminatory policies or systems. Despite their shortcomings, these findings elevate the need for better understanding of how, and why, minority status is related to health outcomes—and how it impacts health care and quality measurement.

## Conclusion

Although minority stress, resilience, epigenetics, and life course concepts undergird health disparities, there are evidence gaps in how they relate to and explain the role of minority status in observed disparities. There are, however, opportunities to track emerging research on these concepts to gain understanding of underlying mechanisms and factors that confer advantage or disadvantage on health and health disparities. The shift to value-based payments provides impetus for designing, testing, and paying for care that reduces minority stress, targets risks early in the life course, and builds resilience.

## Abbreviation Used

DoHAD: Development Origins of Health and Disease

## Appendix

**Appendix Table A1.** PubMed Search Terms

Search topic	Search terms/keywords	Gross hits (full text)	Appended searches^*^/gross hits
Epigenetics	“epigenomics” OR “epigenetics” OR “epigenesis, genetic/genetics” OR (“epigenesis” AND (“genetic” OR “genetics”) OR “genetic heterogeneity”) OR “epigenesis, genetic/physiology” OR (“epigenesis” AND (“physiology” OR (“genetic” AND “physiology”))) OR “epigenomics/methods” OR “epigenomics/trends” OR (“epigenomics” AND “trends”) OR “DNA Methylation” OR “DNA methylation epigenetics” OR (“DNA methylation” AND “epigenetics”) OR “histone modification” OR “imprinting” OR “noncoding RNA”	862	AND race/ethnicity string—198
			AND race/ethnicity OR Socioeconomic Status string AND health outcomes string—9
			AND socioeconomic status string—16
			AND Health outcomes strings—54
			AND race/ethnicity string AND Targeted health outcomes strings—NA
			(…) AND Cardiovascular disease—29
			(…) AND Pre-term Birth and Low Birth Weight—2
			(…) AND Metabolic disease OR Diabetes—22
			(…) AND Mental Health—14
			(…) AND Chronic Disease—9
Minority stress	“minority stress” OR (“minority” AND “stress”) OR “minority stress theory” OR (“minority” AND “stress theory”) OR “minority stress model” OR (“minority” AND “stress model”) OR “stress, psychological/complications” OR “stress, psychological/epidemiology” OR “stress, psychological/ethnology” OR “perceived discrimination” OR ((“perception” OR “perceived”) AND (“discrimination” AND “psychology)” OR “discrimination (psychology)” OR “discrimination”)	522	AND race/ethnicity string—57
			AND race/ethnicity string OR Socioeconomic status AND health outcomes string—40
			AND Socioeconomic status string—58
			AND Health outcomes strings—113
			AND race/ethnicity OR SES string AND Targeted health outcomes strings—N/A
			(…) AND Cardiovascular Disease—22
			(…) AND Pre-term Birth and Low Birth Weight—1
			(…) AND Metabolic disease OR Diabetes—10
			(…) AND Mental Health—24
			(…) AND Chronic Disease—8
Resilience	“stress-inoculation” OR “stress resilience” OR (“stress-inoculation” AND “resilience”) OR “resilience” OR “psychological resilience” OR (“psychological” AND “resilience”)	248	AND race/ethnicity string—17
			AND Socioeconomic status string—27
			AND Health outcomes strings—71
			AND (race/ethnicity OR Socioeconomic status string) AND health outcomes string—20
			AND older adults string—46
			AND race/ethnicity OR Socioeconomic status string AND older adults string—8
			AND race/ethnicity string AND Targeted health outcomes strings—N/A
Life course	“life course” OR “life courses” OR “life course theory” OR (“life course” AND “theory”) OR (“life course” AND “epidemiology”) OR “life course approach” OR “life course perspective” OR (“life course” AND “perspective”) OR “life change events”	459	AND race/ethnicity string—35
			AND (race/ethnicity string OR Socioeconomic status string) AND health outcomes string—41
			AND Socioeconomic status string—77
			AND Health outcomes strings—120
			AND race/ethnicity string AND Targeted health outcomes strings—N/A

^*^ See “Appendix Table A2. Appended PubMed Search Terms.”

**Appendix Table A2.** Appended PubMed Search Terms

Search topic	Search terms/keywords
Minority group, race/ethnicity	race OR “ethnicity” OR (“race” AND “ethnic”) OR “racial” OR “ethnic” OR “race disparity” OR “racial disparity” OR “ethnic disparity” OR (“Black” OR “African-American” OR “African American” OR “Asian” OR “American Indian” OR “Alaska Native” OR “Hispanic” OR “Latino” OR “Latina” OR “Pacific Islander” OR “Native Hawaiian” OR “minority group” OR “minority status” OR “socioeconomic health” OR (“health” AND “socioeconomic”) OR “socioeconomic risk factor” OR (“socioeconomic” AND “risk factor”) OR “socioeconomic differences” OR (“socioeconomic” AND “differences”) OR “social distribution” OR “social environment” OR (“social” AND (“distribution” OR “environment”)) OR “residence characteristics” OR “neighborhood attributes”
Socioeconomic factors	“socioeconomic health” OR (“health” AND “socioeconomic”) OR “socioeconomic risk factor” OR (“socioeconomic” AND “risk factor”) OR “socioeconomic differences” OR (“socioeconomic” AND “differences”) OR “social distribution” OR “social environment” OR (“social” AND (“distribution” OR “environment”)) OR “residence characteristics” OR “neighborhood attributes”
Health outcomes	“Health outcomes” OR (“health” AND “outcomes”) OR “health outcomes disparities” OR (“health” AND “outcomes” and “disparities”) OR “health status” OR (“health” AND “status”) OR “health status disparities” OR (“health” AND “status” AND “disparities”)
Older adults	(“Medicare”[MeSH Terms] OR Medicare [Text Word]) OR “older patients”[All Fields] OR “older adults”[All Fields] OR “older aged”[All Fields] OR “over 65”[All Fields] OR ((“aged”[MeSH Terms] OR Aged [Text Word]) AND (“adult”[MeSH Terms] OR adult [Text Word])) OR (“aging”[MeSH Terms] OR Aging [Text Word]) OR “aged”[MeSH Terms] OR Geriatric [All Fields]
Targeted health outcomes—cardiovascular diseases	(“cardiovascular diseases”[MeSH Terms] OR (“cardiovascular”[All Fields] AND “diseases”[All Fields]) OR “cardiovascular diseases”[All Fields] OR (“cardiovascular”[All Fields] AND “disease”[All Fields]) OR “cardiovascular disease”[All Fields])
Targeted health outcomes—metabolic diseases OR diabetes	(“metabolic diseases”[MeSH Terms] OR (“metabolic”[All Fields] AND “diseases”[All Fields]) OR “metabolic diseases”[All Fields] OR (“metabolic”[All Fields] AND “disease”[All Fields]) OR “metabolic disease”[All Fields]) OR (“diabetes mellitus”[MeSH Terms] OR (“diabetes”[All Fields] AND “mellitus”[All Fields]) OR “diabetes mellitus”[All Fields] OR “diabetes”[All Fields] OR “diabetes insipidus”[MeSH Terms] OR (“diabetes”[All Fields] AND “insipidus”[All Fields]) OR “diabetes insipidus”[All Fields])
Targeted health outcomes—mental health	(“mental health”[MeSH Terms] OR (“mental”[All Fields] AND “health”[All Fields]) OR “mental health”[All Fields])
Targeted health outcomes—pre-term birth and low birth weight	(“premature birth”[MeSH Terms] OR (“premature”[All Fields] AND “birth”[All Fields]) OR “premature birth”[All Fields] OR (“pre”[All Fields] AND “term”[All Fields]) OR “pre term”[All Fields]) AND (“parturition”[MeSH Terms] OR “parturition”[All Fields] OR “birth”[All Fields]) OR “infant, low birth weight”[MeSH Terms] OR (“infant”[All Fields] AND “low”[All Fields] AND “birth”[All Fields] AND “weight”[All Fields]) OR “low birth weight infant”[All Fields] OR (“low”[All Fields] AND “birth”[All Fields] AND “weight”[All Fields]) OR “low birth weight”[All Fields]
Targeted health outcomes—chronic disease	(“chronic disease”[MeSH Terms] OR (“chronic”[All Fields] AND “disease”[All Fields]) OR “chronic disease”[All Fields])
